# Review of the Medical Department of the East India Company

**Published:** 1830-07-01

**Authors:** 


### Review of the Medical Department of the East India Company.

For several years past we have witnessed, with pain, the depressed state of the medical profession in this country. The ranks of medical society are so crowded, that the professors of a noble art are driven, by actual want, to the most disingenuous devices—to the necessity of stabbing one another's reputation, and preying on the public at large! There can be no doubt that this redundancy in our ranks is only part and parcel of that general redundancy in all departments of civilized life. The law has felt it for some time, and a heavy prohibitory fine is about to be levied on all who enter that learned profession. We do not suppose that such a measure is practicable in medicine, though we are far from thinking that it would be prejudicial to the profession at large, or to the public generally, however hard it might bear on individuals, or however it might press on humble merit. The fact is, that we stand more in need of liberal education and honorable conduct, than of talent without these qualifications. There is little danger of want of ability in the rising generation of the profession; but there is every reason to apprehend a dreadful deficiency of liberality and high-mindedness—partly from the crowded state of medical society—partly from the leaven of corruption and vulgarity which is so plentifully disseminated in the present day.

We have now, however, to draw attention to a great outlet and field for medical talent, which has hitherto attracted a crowd of competitors, but which is likely to prove a source of dreadful disappointment to great numbers of our medical and surgical aspirants. The amplitude of our East-India possessions, and the encouragement which has been held out for medical students to embark for those burning climes, there to spend their lives under the confident expectation, or even assurance, that a youth, or rather a "*life* of labour" would be crowned with an "age of ease," have caused the portals of the Temple, in Leadenhall Street, to be almost choaked with candidates for obtaining the golden fruit of the pagoda-tree. In the best of times, this attractive fruit was dearly purchased at the expense of banishment, sickness, and premature old age, if not untimely death. Now, however, the fruit is taken away, and it appears that the medical practitioner, in the service of our Honorable East India Company, is estimated somewhat under a butler in London! By the said Company a man is considered as far inferior to a horse—and consequently a surgeon is subordinate to a black-smith! Our rising choler has been checked, indeed, by one redeeming principle or sentiment, which the Company have lately evinced, and which must command the respect and esteem of the world. Wisely considering that the life of man is transitory in this sublunary scene—that it is bounded to three score years and ten, at the most; and, *in their service*, to little more than half that duration, while the soul flourishes in eternal youth, beyond the boundaries of matter, they have very properly estimated the surgeon, who has charge of this vile body, as a cypher, while the chaplain, who directs the etherial spirit to its future abode of felicity, is considered as a most important personage, little inferior to the Pope, or even to St. Peter, with the keys of Heaven in his hands! These observations will scarcely be believed, or even understood, till the following document is perused:—and we deem it a duty, which we owe to our profession, and to the fathers of medical youths, who may be fondly dreaming of golden settlements for their sons beneath the Torrid Zone, to publish this memorial entire, in order that they may know the extent of misery which they are unconsciously preparing for their offspring.

### THE MEMORIAL

### Of the Medical Officers of the Bengal Presidency, whose Signatures are hereunto annexed, to the Chairman, Deputy Chairman, and Directors of the Honorable the East India Company, &c. &c. &c.

We, the undersigned Medical Officers of the Bengal Presidency, most humbly and respectfully solicit the attention of your Honorable Court to the existing state of the medical department of this country, which, by the recent abolition of the medical contract hitherto in force, is so entirely changed in its character and provisions, and the interests of your Memorialists are so deeply affected thereby, that they feel themselves warranted in submitting this detailed statement of their grievances to the favourable consideration of your Honorable Court, in the anxious hope that they may be speedily relieved from those just, and urgent grounds of complaint, under which they at present suffer.

The emolument derived from the late medical contract, though perhaps objectionable in its principle, nevertheless afforded to a certain extent an average remuneration to Medical Officers, proportionate to the duties performed, the abolition of which, both in its present effects and its future consequences, entails on your Memorialists, and more especially on the junior grades, a state of embarrassing poverty and ultimate ruin, which they persuade themselves could never have been contemplated by your Honorable Court as a result of the orders of the Governor General in Council, bearing date 29th November, 1828.

Your Memorialists entered the service of the Honorable Company confidently relying on the permanency of those advantages which were enjoyed by the medical staff of this presidency, and of which, from whatever source derived, your Memorialists could never have contemplated any diminution, as even under their operation the average income, received by Medical Officers could be considered a bare equivalent for the laborious and important duties assigned to them.

Previous to entering into those details which they consider essential to a due estimate of their claims by your Honorable Court, they earnestly solicit a full restoration of the average income they formerly enjoyed, and of which, on the principles of equity, they humbly conceive they ought never to have been deprived. Your Memorialists having thus briefly submitted the sum of their petition, and the grounds on which they justify their claims, feel themselves called upon to state, for the information of the Honorable Court, the following aggregate of facts, as demonstrative of that inferiority, in point of respectability and ultimate provision, which they have at all times suffered, in comparison with every other department of your public servants.

The subjects which your Memorialists beg to press on the attention of the Honorable Court, is the great length of time indispensable to the attainment of their professional education and the heavy attendant expenses which equal, if not exceed those incurred in the acquisition of any other liberal profession; and, with reference to such expenses, your Memorialists assumed, on premises acknowledged to be indisputable, the well-grounded expectations of an adequate return, in whatever channel they might have directed their talents and exertions, but which your Memorialists most deeply lament were never realized, and are now totally annihilated.

The comparatively advanced period of life (that of 23 to 25 years of age) at which your Memorialists enter the service, is the unavoidable result of the time employed in the course of education, as above stated; a consequence of the utmost moment to your Memorialists, as involving the rate of promotion; since by the operation of the orders above referred to, combined with the numerical strength of the medical list, your junior assistant surgeons are entirely shut out from the prospects of attaining the rank of surgeon, under a period of from 17 to 20 years, during the whole of which time, it is absolutely impossible for them, in the receipt but of a bare subsistence, to support that relative rank in society they have hitherto maintained, far less to procure those ordinary comforts of life, which habit, climate, and education have rendered indispensable.

From the facts herein adduced, it necessarily follows that a medical officer, should he even live to attain the rank of a surgeon, will have reached the age of

43 or 45 years, when he may avail himself of the miserable pension of £191 per annum, a sum utterly inadequate to support him in the rank of a gentleman and totally disproportionate to his past services.

The prospects of your Memorialists, even when advanced to this rank, though placed on a somewhat fairer scale of remuneration, will appear but little improved, when the numerous contingent disadvantages are duly considered;—for it will be evident to your Honorable Court that thus, after 20 years service, are they, for the first time, enabled to save a fraction of their allowances, to defray either the expenses of a furlough to Europe, or to add to their future provision; while it must be borne in mind, that no individual can pass 20 years continuously in a tropical and unhealthy climate, without materially suffering in health and constitution; and this more particularly applies to your Memorialists, exposed as they necessarily are, in all seasons and under all circumstances, not only to the fatigues and duties of the military profession, but to the labour, trouble, and anxiety of their own.

Adverting to their next higher grade of superintending surgeon, a rank not heretofore attained under an average period of 16 years more, your Memorialists beg to observe that the obstacles to promotion already mentioned will, in future, defer that prospect to an almost indefinite period, certainly to one by which they will have attained the age of 60 years;—and even then, after nearly 40 years' service, they cannot claim the trifling pension of that rank, till they shall have served an additional two years in that situation, an act of illiberality and injustice inflicted on no other department of your public service.

On a review of the facts above stated, it must necessarily be inferred, that your Memorialists can never entertain the most distant hope of reaching the highest grade of your medical service, viz. the Medical Board, under four and forty years, and at the advanced age of seventy; a period of human life far exceeding that generally attained in this or any other country.

The deplorable situation of your Memorialists must be sufficiently apparent to your Honorable Court, from the general outline of their grievances above stated; and they persuade themselves that they do not incur the charge of presumption, in preferring the question, with all humility and respect, what possible contingency, in their case, could have caused, much less perpetuated or extended, so total an absence of consideration for their character and services as a comparative view of their condition cannot fail to evince.

Possessing an imaginary rank in the army, without enjoying any of those privileges or prospects to which the real possessors of such rank are entitled, your Memorialists are, from the exclusive nature of their profession, necessarily ineligible to those various places of rank and emolument, which are open to the purely military officer of whatever grade; and, so far from receiving any equivalent for those disabilities, in any corresponding situations in their own department, their present and future provisions are totally disproportionate to their actual or eventual services, while it cannot be supposed that a medical officer can look forward with satisfaction to the retiring pension of £191 12*s.* per annum, whether he may have served 20 or 36 years—of £300 per annum, after 36 to 44 years—or of £500 per annum, after 44 to 47 years, at the respective ages of 43 to 45 years, for the first rate of pension—59 to 61, for the second—and of 67 to 71, for the third; your military officers, in the mean time, enjoying a superiority of advantage in this respect, in the following rates.

1st. The military officer may retire in 25 years, at the age of 41, upon £273 per an.
2d. In 28 years, at the age of 44........................ upon £365.
3d. In 33 years, at the age of 49........................ upon £1000.

Contrasts so forcibly exemplified in themselves, as to require little comment in further explanation of the great inferiority of your medical service. And hence it appears how totally suppositious are the advantages held out to your Memorialists on entering the service by the regulations in the East India Directory at Home, under the sanction of your Honorable Court; the plainest inference deducible from which is the possibility of the ranks of superintending surgeon and of member of the Medical Board being attained in the periods therein mentioned, viz. 17 to 20 years; results which have never yet or ever can obtain in the medical department of this presidency, where the ratio of promotion to the highest situation is in the proportion of 1 to 130 of the total establishment.

Nevertheless your Memorialists disclaim the most distant imputation of invidiousness at the better fortune of their military brethren, or of any other branch in your honorable service ; but they have deemed it necessary to particularize the above contrasts, in order to shew, beyond question, their real state of comparative inferiority, in relation to other departments ; in further illustration of which fact, they beg to advert to your military chaplains, who enter your service at the corresponding ages of your Memorialists, (without having incurred one half of that expense necessary to the medical education,) and whose income, from the day of their arrival in India, exceeds that of your military servants, even after a period of 20 years service, independent of the incomparable advantages the former enjoy of returning to Europe, after the short period of 15 years, on the pension of lieutenant colonel, viz. £365 per annum.

It would be illiberal on the part of your Memorialists, to draw any comparison between themselves and your ecclesiastical servants ; but, on the score of public utility, arduous duty, general information, and professional character, it cannot be shewn that the latter possess any claim on your Honorable Court for a scale of remuneration so superior to that granted to your Memorialists.

In proving this their abject condition, your Memorialists use this line of argument, not, as above declared, from feelings of envy: but purely and honestly to draw your attention to the urgent necessity of its speedy amelioration ; and, in this spirit, they beg to adduce a further instance of their merits and claims being totally overlooked by your Honorable Court, in that countenance and provision you have, to the ignominious prejudice of your medical establishment, vouchsafed to a class of persons lately introduced into your service, under the denomination of veterinary surgeon.

Your Memorialists, in no degree doubting the utility of the veterinary art, or indeed of any other contingent branch of knowledge, studiously avoid entering into any comparative enquiry as to its nature and character, or the time and expense necessary to its attainment ; but it is indeed, with feelings of humiliation and regret, that, for the first time on record, they should be held forth to the world by your Honorable Court, as less worthy of remuneration for the care and responsibility incurred by them for the preservation of the health and lives of their fellow creatures (your Honorable Company's lieges) than those intrusted with the lives and health of the horses in your Honorable Company's army ; a feeling so derogatory to human nature generally, and more especially, in its application to the character of your Memorialists, that they persuade themselves your Honorable Court could only have been betrayed to evince it by some informality or inadvertence in the routine of official business.

The following Table, extracted from the orders of your Honorable Court, bearing date the 27th October, 1827, and from the General Orders of your supreme Government, dated 9th February, 1828, exhibit the ignominious contrast above alluded to.

| Pay and Allowances of Veterinary Surgeons under three Years Service for Charge of 400 Horses, the strength of a Cavalry Corps | Per Mensem. | | | Pay and Allowances of Veterinary Surgeons above three Years Service for Charge of 400 Horses. | Per Mensem. | | |
|---|---|---|---|---|---|---|---|
| Pay .................... | 97 | 6 | 5 | Pay ...................... | 121 | 12 | 0 |
| Batta .................... | 121 | 12 | 0 | Batta .................... | 121 | 12 | 0 |
| Gratuity .................. | 24 | 0 | 0 | Gratuity .................. | 24 | 0 | 0 |
| Tentage .................. | 50 | 0 | 0 | Tentage .................. | 50 | 0 | 0 |
| Horse Allowance ........ | 47 | 13 | 3 | Horse Allowance ........ | 47 | 13 | 3 |
| Pallanqueen Allowance.... | 30 | 7 | 0 | Pallanqueen Allowance.... | 30 | 7 | 0 |
| Total Sonat Rupees .. | 371 | 6 | 8 | Total Sonat Rupees.. | 395 | 12 | 3 |

| Pay and Allowances of an Assistant Surgeon for the sole Medical Charge of 800 Officers and Men, exclusive of their Families, Servants, &c. | Per Mensem. | | | Pay and Allowance of an Assistant Surgeon for Medical Charge of 800 Officers and Men at an Half Batta Station. | Per Mensem. | | |
|---|---|---|---|---|---|---|---|
| Pay | 60 | 14 | 0 | Pay | 60 | 14 | 0 |
| Captains full Batta | 182 | 10 | 0 | Captains half Batta | 91 | 5 | 0 |
| Gratuity | 24 | 0 | 0 | Gratuity | 24 | 0 | 0 |
| Tentage | 50 | 0 | 0 | Half Tentage | 25 | 0 | 0 |
| Palanqueen Allowance | 30 | 0 | 0 | Pallanqueen Allowance | 30 | 0 | 0 |
| | | | | House Rent, if not provided with Quarters | 30 | 0 | 0 |
| Total Sonat Rupees.. | 347 | 8 | 0 | Total Sonat Rupees.. | 281 | 3 | 0 |

Your Memorialists beg to observe that, from the constitution of the medical department in this country, it appears to have been assumed that they were not naturally liable to that decay of health and constitution consequent on length of service in a tropical and unhealthy climate, in common with their countrymen and fellow servants; since no provision for such contingency has ever been recognized for that department, a subject to which your Memorialists earnestly crave the consideration of your Honorable Court.

Your military servants, in the junior ranks of lieutenant and captain, when disabled from active military duties by sickness or broken health, are eligible to various local appointments, yielding a comfortable subsistence, in the invalid battalions, garrisons, paymasterships of pensions, and several others which it is unnecessary to detail; while, in the higher ranks of major and lieutenant colonel, the command of provincial battalions, invalid tannacks, local corps, invalid corps, and garrisons, not only affords them a handsome independence, but, in fact, equals in many instances, the allowances of those in the line, without entailing on the one the expenses or important duties of the other; while the maximum of pension granted to a medical officer, who may have served your Honorable Company from 36 to 44 years, is only 300 sonat rupees per month.

The non-existence of such provision in the medical department, is the more to be regretted, not only as it now or eventually affects those who from misfortunes or embarrassments may never have the means of returning to Europe, but more especially as it involves consequences injurious to humanity by your medical servants being compelled to remain in the performance of important duties, to which from broken health and constitution they are unequal, and from the discharge of which it cannot be doubted they would gladly retire on receiving an adequate provision in the invalids establishment, without which it would be unreasonable to expect they would willingly relinquish their only means of support.

The principle of an adequate provision on a footing of respectability and comfort for all grades of your military servants when worn out and incapacitated for active duty by climate and length of service, has been fully recognized and long acted upon by your Honorable Court, not only in regard to your European, but to your native servants also, and your Memorialists trust such an exception in their case will no longer be upheld; and that your Honorable Court will sanction such arrangements in the fixed allotment of the medical duties of garrisons, civil stations, and invalid pay, as would yield a comfortable provision for those members of the medical profession labouring under the above-mentioned circumstances, a measure which, on the score of promotion, would prove at the same time as beneficial to the medical, as its operation proves to be in the purely military department.

In the assimilation of rank of the officers of the army with those of His Majesty, your Memorialists have, in the grades of assistant surgeon and surgeon, the corresponding nominal ranks assigned them of lieutenant and captain; but this correspondence of rank in the two medical departments ceases with reference to the superior grades, and hence your Memorialists are excluded by the regulations of your service from that distinction and respectability in the army and corresponding rate of income enjoyed by His Majesty's medical staff; an anomalous privation to which your medical servants are alone subjected.

This will be obvious to your Honorable Court, on reference to the Pay-Table of the Medical Staff in His Majesty's Service, by which it appears that a surgeon, after 14 years' service, receives 14*s*. 3*d*. per diem. A surgeon after 18 years' service receives 18*s*. 10*d*. per diem. In the next rank, that of physician to the forces, 19*s*. per diem. Of deputy inspector of hospitals, 1*l*. 3*s*. 9*d*. per diem. Of inspector of hospitals, 1*l*. 17*s*. per diem. Of principal inspector of hospitals, 1200*l*. per annum. Of director general of hospitals, 2000*l*. per annum.

The above scale of remuneration strongly exhibits the unmeasurable superiority enjoyed by the medical department of His Majesty's service, when contrasted with that of your Honorable Company; while the numerous other advantages attendant on these medical grades, (advantages not existing in your honourable service,) render the contrast still more unfavourable to your Memorialists.

The most general and important of these advantages consist in the medical officers of His Majesty's army being chiefly employed in, and deriving their emolument from, numerous medical situations distributed throughout their native country, as hospitals, garrisons, and depôts;—head quarters of military and recruiting districts, those of the ordnance department, and various others unnecessary to mention, exclusive of the many medical staff appointments in His Majesty's colonies, not only in the West Indies, but in the Mediterranean, the Cape of Good Hope, the Mauritius, Ceylon, New South Wales, and the Canadas, all of which have corresponding local advantages, or contingent as comfortable quarters, free of expense, wine free of duty, coals, candles, forage, general cheapness of living, and, in most parts, an equalization of currency, and by exchanging appointments with their professional brethren in Britain, they may continue to enjoy these peculiar advantages, the absence of which in your service entails on your Memorialists a state of perpetual exile from their home and country, a sacrifice required of them without any corresponding or equivalent benefits being conferred.

As a practical instance of the want of corresponding rank in the army, we beg to adduce the case of Dr. Burke, His Majesty's inspector of hospitals in Bengal, who, from his corresponding rank of colonel in the army, receives the same amount of salary as the members of your Medical Board, and for this reason shared in the distribution of prize money for the capture of Bhurtpore with the officers of that rank, while Mr. Superintending Surgeon Reddil, the senior in rank of all the Company's medical officers, present on that occasion, shared only with majors after a period of 36 years' service, and in the performance of similar duties; an unjust distinction, not only implying an inferiority of merit, but entailing a pecuniary loss to your Memorialists on a casual occasion only once to be expected in the longest course of military service, and consequently the more to be valued.

Your memorialists have yet to advert to a resolution of your Honorable Court published by your Governor General in Council, in military orders, No. 254, of the 29th November last, equally afflicting your Memorialists in common with your other military officers, by subjecting them to a loss of half batta, at the four principal lower stations of the army of this presidency;—a measure they cannot look upon without alarm and dismay, as they consider it a direct infringement of that implied understanding on which they entered your service; a breach of contract, if once recognized, that may be extended ad libitum; for on the same principle, your Honorable Court may withhold the remaining portion of their allowances, either wholly or in part, or even the amount of their retiring pensions; and, in respect to themselves individually, they cannot understand an assumed principle in the same orders, wherein, with advertence to the abolition of the medical contract, the full batta of captains and majors is granted respectively to assistant surgeons and surgeons, as compensation or in lieu of the said contract, while that very compensation is entirely neutralized by another provision of the same order, thus depriving your Memorialists of all indemnification whatever.

The pay-tables of 1796, fixing on a permanent scale the allowances of the Bengal army, were not adopted till after a full discussion of their provisions and ultimate sanction of His Majesty's Ministers; consequently, your Memorialists believe, with due deference to your Honourable Court, that no authority, short of that which established the scale of their pay and allowances thirty years ago, can now diminish them a single fraction, and that they are legally secure from any inroad on their income, till their claims are again discussed in the British Parliament, and settled by its decision under the control of the officers of the crown; neither can they imagine that a power temporarily delegated by the Sovereign to a corporate body can diminish the pay of the Officers of a large portion of the integral army of the state (which the Native army of India undoubtedly is) when a like authority is withheld from His Majesty in regard to the army of England.

But waving for the moment all considerations of equity, justice or policy, no stronger argument can be adduced against the diminution of our allowances by the introduction of Half Batta, than that furnished by your Honorable Court; and your Memorialists respectfully solicit reference to your military letter addressed to the Government of Fort St. George, bearing date the 15th September, 1809, where, in the 65th Para, the injustice of such diminution is fully recognized, as will appear by the following extract. "The persons nominated to civil and military employment have entered the services perfectly aware of these inequalities, and are therefore not entitled to expect they should be afterwards removed," and this idea of a general equalization of allowances throughout the three Presidencies, so decidedly and justly resisted at that time by your Honorable Court, as a direct breach of the covenant and mutual stipulations, under which your civil and military officers were engaged, has now, by the enforcement of the late order of your supreme Government, No. 254, of 29th November last, been fully acted upon; exhibiting an alteration in the sentiments and reasoning of your Honorable Court, so totally at variance with those clearly expressed in 1809, and so injurious in its effects to the interests of your Memorialists, as can be justified by no plea of expediency and necessity, nor reconciled to the most ordinary principle of good faith :—while it is impossible to characterize the strange anomaly existing in the said order, where, by the privation of one half of their Batta, the nominal equivalent granted to your Memorialists as a compensation for the loss of the medical contract is withheld from them, even in this, the very order announcing it; and if the admission of your Honorable Court, in the 64th Para, from the same letter, "that the style of living among Europeans has gradually adapted itself to the scale of income," were tenable and recognized in 1809, how many thousand times more urgent does it become in its application to the circumstances of the present period, when the actual expenses of the most common necessaries of life, of servants, &c. have trebled or quadrupled. The enormous depreciation of the currency and consequent loss of exchange, losses incurred in houses by abolition of old or formation of new military stations (for which, to whatever amount, no compensatory allowance is ever granted,) duties augmented on articles of every description, and especially the ruinous expenses of travelling, to which all military officers are exposed from the greater extension of territorial possession and increased distance of stations, but to which your Memorialists, as belonging to the whole army, are peculiarly subjected, being frequently called upon, on the shortest notice, to pass from one extremity of the Bengal Presidency to the other, to the detriment of their health, and to the total ruin of their finances; disadvantages which, when combined and maturely considered, not only oppose most forcibly every idea of reduction, but most imperatively call for an augmentation of their allowances.

To these natural feelings of dissatisfaction which your Memorialists have ventured to express, should it be answered that their further services are not compulsory, and that they are at liberty to withdraw them, we beg to state to your Honorable Court, that such an assumption is totally erroneous, as they have neither realized the amount of the heavy expenses incurred by their early education, &c. or even the means of returning to Europe; but imagining, for an instant, they had done so, what indemnification would such return be to them for the sacrifice they irrevocably made in entering your service, when, relying on the integrity and justice of your Honorable Court, and on the permanency of the advantages held out to them, they left their native country, their families and connexions, under whose countenance and by whose assistance, they would have been enabled, at that time, to have established themselves in public life, an opportunity now lost to them for ever—which circumstance alone will doubtless induce your Honorable Court favourably to listen to and speedily to grant the prayer of your Memorialists.

In further illustration of the systematic neglect, and of the very subordinate character in which the medical department of this Presidency is held, may be adduced the total want of patronage in the members of the Medical Board, whose professional abilities, experience in the country, and knowledge of individuals, would, it is supposed, have pointed them out as the most proper distributors of it; but superintendence is rendered nearly nugatory by their inability to reward merit, or encourage talent, thus compelling your Memorialists to court the patronage and support of the military officers commanding Divisions, Brigades, &c. and more especially of the Adjutant General of the army, whose abilities, great and transcendant in their military capacity, can never be increased by a dubious judgment of the comparative merits, and qualifications of medical officers, the operation of which system is highly detrimental to the service generally, but particularly so to the department of your Memorialists, *since a struggle for interest and favour supersedes the honest emulation for fame, rank, or emolument.*

That this department of your service has been ever and only considered as an inferior and degraded appendage to your civil and military establishments, will appear from the fact that, in the arduous and harrassing campaigns in which your Bengal army has been engaged during the last fifteen years (with two individual exceptions) no public acknowledgement, of any kind, has ever been paid to its members, however great or meritorious their services—however anxious and responsible their duties—to whatever severe and painful trials their feelings may have been subjected! To the due performance of the duties assigned to them, duties in which all personal feelings are merged in the alleviation of suffering humanity, there is a degree of mental satisfaction which no contempt or neglect can diminish, and the enjoyment of which, is the only reward of your Memorialists; but as men of honorable feeling and liberal education, they are not insensible or culpably indifferent to the approbation of their superiors and associates, whose opinion of them is generally formed, not on their merits (which they cannot appreciate) but on their rank in public estimation;—and, in confirmation of these ignominious privations and invidious distinctions having been long and acutely felt by your professional servants, may be incidentally mentioned a well attested fact, *that no medical officer in this Presidency, for the last 20 years, has ever sent his son to India in a medical, though many have done so in a military capacity!!*

From this detailed statement of their grievances and hardships, and of the disqualifications and disadvantages they suffer, it will be manifest to your Honorable Court, that your Memorialists are an ill-paid, neglected, and degraded class of public servants, and they appeal to the liberality, justice, and honor of the Court, that, when this their true and deplorable condition is examined and reflected on, such a revision may be made in the whole system of the medical department, as will place your Memorialists in that rank in society to which they are entitled, and raise, their allowances to a just scale of indemnification for their service and sacrifices; and they trust they do not incur the charge of presumption, or of assuming the language of dictation in suggesting the following as a scale consonant to these intentions, and which, in their humble judgment, is the least that can be considered as a fair and adequate remuneration.

1st. That the junior grade be divided into three classes according to length of service, with proportionate allowances in each; that assistant surgeons (3d class) from their entrance into the service to the expiration of five years, receive each in all situations while actually holding any medical charge, the sum of 400 rupees per mensem, subject to a monthly deduction of 100 rupees previous to his joining such charge, or when absent on private affairs or medical certificate.

2d. That from and after five years service assistant surgeon's (2d class) receive for the next five years ensuing, the sum of sonat rupees 600 per mensem, subject to a similar deduction.

3d. That from and aften ten years service, assistant surgeon's (1st class) receive the monthly sum of 800 rupees, till their promotion to the rank of surgeon, subject to the same deduction under similar circumstances.

4th. That a regimental surgeon holding medical charge of a Native cavalry, infantry, or artillery corps, receive in all situations, 1000 rupees per mensem, and when in charge of an European corps, an additional 260 rupees, or a total of 1260 a month, subject, in both instances, to a deduction monthly of 200 rupees when absent on leave, &c.

5th. That an intermediate medical grade be established between the regimental surgeon and superintending surgeon, corresponding in rank to that of deputy inspector in His Majesty's army, under the designation of "Cantonment Staff Surgeon," for the duties of the head quarters of military divisions, and with a proportionate salary.

6th. That the pay and allowances of superintending surgeons remain as at present, and that members of the medical board receive sicca rupees 3000 per mensem; and in reference to the above scale, that the furlough pay, allowed in each rank be as follows;—

To assistant surgeons, if compelled to return to Europe on account of ill-health, at any time previous to ten years actual service in India £150 per annum. And after ten years until promoted to the rank of surgeon £200 per annum. Regimental surgeons, and all other classes £300 per annum; for the established period, viz. three years.

Considering their duties equally important and their services equally valuable as those of any other of your public servants, your Memorialists submit that the amount of their retiring pensions, now so miserably small and inadequate, should be put on a fair and full equalization with those of military officers and chaplains, a claim to which on the bases of equity, justice, and expediency, they conceive themselves fully entitled, but of which they have been hitherto deprived, and the following scale proportioned to length of service which your Memorialists venture to submit for the approval of your Honorable Court, is neither extravagant in itself, nor when viewed comparatively does it exceed that granted to military officers after a similar period of service.

We therefore entreat your Honorable Court, that a medical officer of whatever grade, should be allowed after a continuous period of service of 17 years, the retiring pension now granted to your military chaplains, and after a period of 25 years £450 per annum; from 25 to 36 years £600 per annum; and from 36 to 44 years £1000 per annum.

Pensions, which, from the great mortality in all classes of your medical servants, can never form any considerable feature of expense to the Honorable Company, as will clearly appear by reference to the total existent amount of retiring pensions to the medical staff of this Presidency, and which pensions, while they indicate a juster appreciation of the merits and abilities of your Memorialists, and secure to them a fair and suitable remuneration, directly conduce to the welfare of your service, by promoting the efficiency and respectability of the medical department, whose high character for intelligence and humanity, under circumstances the most trying, has been ever maintained and acknowledged, not only by your own servants but by the unanimous suffrage of their professional brethren throughout the world.

Your Memorialists therefore conclude their appeal to your Honorable Court by earnestly and anxiously pressing on your notice the urgent and indispensable necessity of immediately relieving them from that state of degradation and poverty under which they now so unjustly suffer,—confidently relying on the justness of their petition and on the liberality of your Honorable Court,

We have the honor to subscribe ourselves,
Your faithful humble Servants, &c. &c.

We have given promulgation to this memorial from a sense of the great hardships and injustice under which our medical brethren of the East now appear to labour—and as a warning to those who may be preparing to embark for that fatal and melancholy climate, under the delusive hope that their services there may be either appreciated or rewarded. For our own parts, we would not send a dog for whom we had any regard, into the medical department of the Company's Service, if that faithful animal were capable of holding a commission under the Rajahs of Leadenhall Street!

The perusal of this memorial is well calculated to call forth feelings not very favourable towards that IMPERIUM in IMPERIO which sits in conclave at home, and dooms its servants to sickness, poverty, and insult, on the swampy banks of the Ganges. Nothing can be more clear than the approaching "decline and fall" of our Indian empire, occasioned or accelerated, perhaps, as in other and more celebrated instances, by corruption or injustice at home!

